# Single‐cell transcriptomic profiling reveals transcription factor‐driven CD14^+^ monocyte programs underlying divergent wound healing outcomes in diabetic foot ulcers treated with Shangke Huangshui

**DOI:** 10.1002/ctm2.70838

**Published:** 2026-11-14

**Authors:** Jue Zhang, Wenyi Cao, Mingxun Luo, Zhimin Gu, Ming Mu, Junjiu Huang, Xiaotong Luo, Aisheng Wei

**Affiliations:** ^1^ The Eighth Clinical Medical College of Guangzhou University of Chinese Medicine Foshan P. R. China; ^2^ Foshan Hospital of Traditional Chinese Medicine Foshan P. R. China; ^3^ Guangdong Provincial Key Laboratory of Pharmaceutical Bioactive Substances, School of Biosciences and Biopharmaceutics Guangdong Pharmaceutical University Guangzhou China; ^4^ MOE Key Laboratory of Gene Function and Regulation, State Key Laboratory of Biocontrol, School of Life Sciences Sun Yat‐sen University Guangzhou China; ^5^ Innovation Centre of the Sixth Affiliated Hospital, School of Life Sciences Sun Yat‐sen University Guangzhou China; ^6^ Department of Gynaecology, Clinical Transformation and Application Key Lab for Obstetrics and Gynaecology, Paediatrics, and Reproductive Medicine of Jiangmen, Jiangmen Central Hospital Affiliated Jiangmen Hospital of Sun Yat‐sen University Jiangmen China; ^7^ Guangdong Institute of Gastroenterology, Biomedical Innovation Centre, The Sixth Affiliated Hospital Sun Yat‐sen University Guangzhou China

Dear Editor,

The complex pathology of diabetic foot ulcers (DFUs) drives high disability and recurrence rates. Although the topical Traditional Chinese Medicine Shangke Huangshui (SKHS) promotes healing, significant therapeutic heterogeneity exists, and its mechanisms remain unclear.^1^ Here, we performed exploratory longitudinal single‐cell RNA sequencing (scRNA‐seq) on peripheral blood mononuclear cells (PBMCs) from DFU patients before and after SKHS therapy, comparing immune profiles between responders (Heal) and non‐responders (Non‐heal). We identified outcome‐associated transcription factor programs in CD14^+^ monocytes. Specifically, a CD14_NKG7 subset with a cytotoxic‐like transcriptional signature and a “high‐efficacy, low‐inflammation” phenotype was associated with inflammation resolution within a critical two‐week window in responders. Conversely, non‐responders exhibited metabolic reprogramming co‐occurring with upregulated drug metabolism and FOS family‐associated inflammation amplification, suggesting potential resistance‐related mechanisms.

We performed scRNA‐seq on PBMCs from five DFU patients (Heal group, *n* = 3; Non‐heal group, *n* = 2) at baseline (0 W), 2 W, and 3 W post‐treatment (Figure 1A; baseline characteristics compared in Table S3; detailed methods in Sections S1.1–1.6). Unsupervised clustering annotated these cells into nine major immune cell types (Figure 1B–D and Figure S1A,B). Cell proportion and Observed/Expected (Ro/e) ratio analyses revealed that CD14^+^ monocytes abnormally expanded in the Non‐heal group, peaking at 2 W (Figure 1E–G and Figure S1C,D). Given that DFU healing correlates with systemic inflammatory status, inflammation score analysis further demonstrated that these Non‐heal CD14^+^ monocytes displayed significantly higher inflammation scores (Figure 1H and Figure S1E).

**FIGURE 1:**
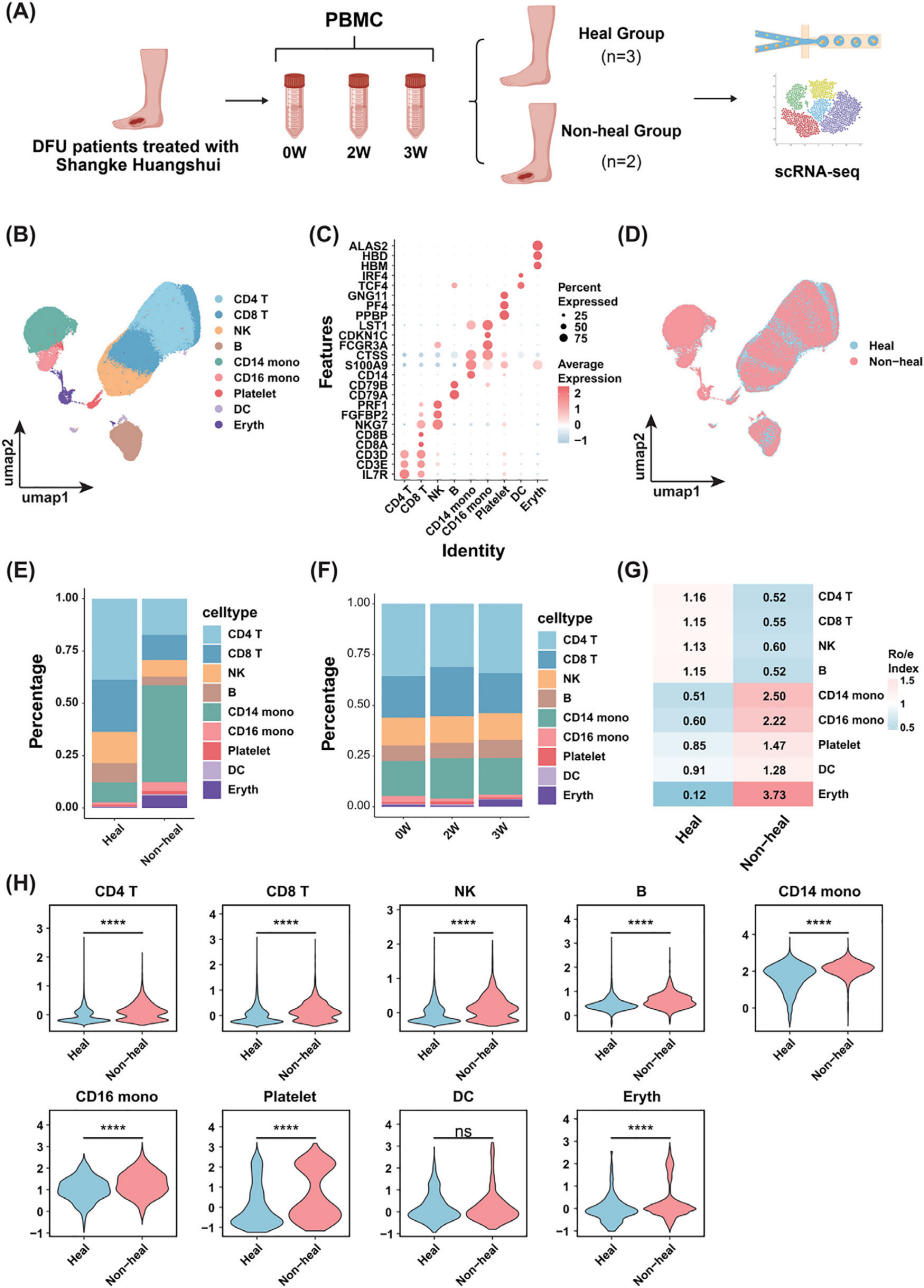
Single‐cell Atlas of peripheral blood mononuclear cells (PBMCs) in diabetic foot ulcers (DFUs). (A) Workflow diagram illustrating the study design and analytical methods. (B) UMAP plot displaying the distribution of different cell populations. (C) Bubble plot showing the expression of characteristic genes across various cell populations. (D) UMAP plot illustrating the cell distribution in the Heal and Non‐heal groups. (E) Stacked bar chart depicting the relative proportion of cell numbers in the Heal and Non‐heal groups. (F) Stacked bar chart showing the relative proportion of cell numbers at treatment time points 0, 2, and 3 W. (G) Ro/e analysis heatmap displaying the expected expression differences of cell populations between the Heal and Non‐heal groups. (H) Violin plot showing the inflammatory functional phenotype scores of each cell population, with statistical analysis evaluating the significance between the Heal and Non‐heal groups.

Sub‐clustering of CD14^+^ monocytes identified six distinct subsets through differential expression analysis (Tables S4 and S5). Combined with canonical markers, these were annotated as: pro‐inflammatory CD14_TSPO and CD14_NLRP3; metabolically active CD14_DPYD‐AS1; CD14_NKG7 with a cytotoxic‐like transcriptional signature; antigen presentation‐related CD14_HLA‐DRA; and CD14_BANK1 (Figure 2A,B). GSVA revealed significant enrichment of metabolic stress and pro‐inflammatory pathways in the Non‐heal group, contrasting with T‐cell receptor (TCR) signalling regulation and antigen presentation in the Heal group (Figure S2A). Consistently, pro‐inflammatory subsets (CD14_TSPO and CD14_NLRP3) predominated in the Non‐heal group, while the CD14_NKG7 subset with a cytotoxic‐like transcriptional signature was enriched in the Heal group (Figure 2C and Figure S2B). Monocle2 pseudotime and CytoTRACE analyses demonstrated a dynamic differentiation trajectory where the Non‐heal‐enriched and Heal‐enriched subsets segregated at opposite ends. CytoTRACE and progenitor scoring confirmed the early differentiation state of CD14_TSPO and CD14_NLRP3 versus the terminally differentiated state of CD14_NKG7 (Figure 2D and Figure S2C–E). Cell proportion analysis showed that the CD14_NKG7 subset was preferentially enriched in the Heal group, peaking at 2 W (Figure 2E), suggesting that it may represent a candidate effector population associated with SKHS response and wound healing.

**FIGURE 2:**
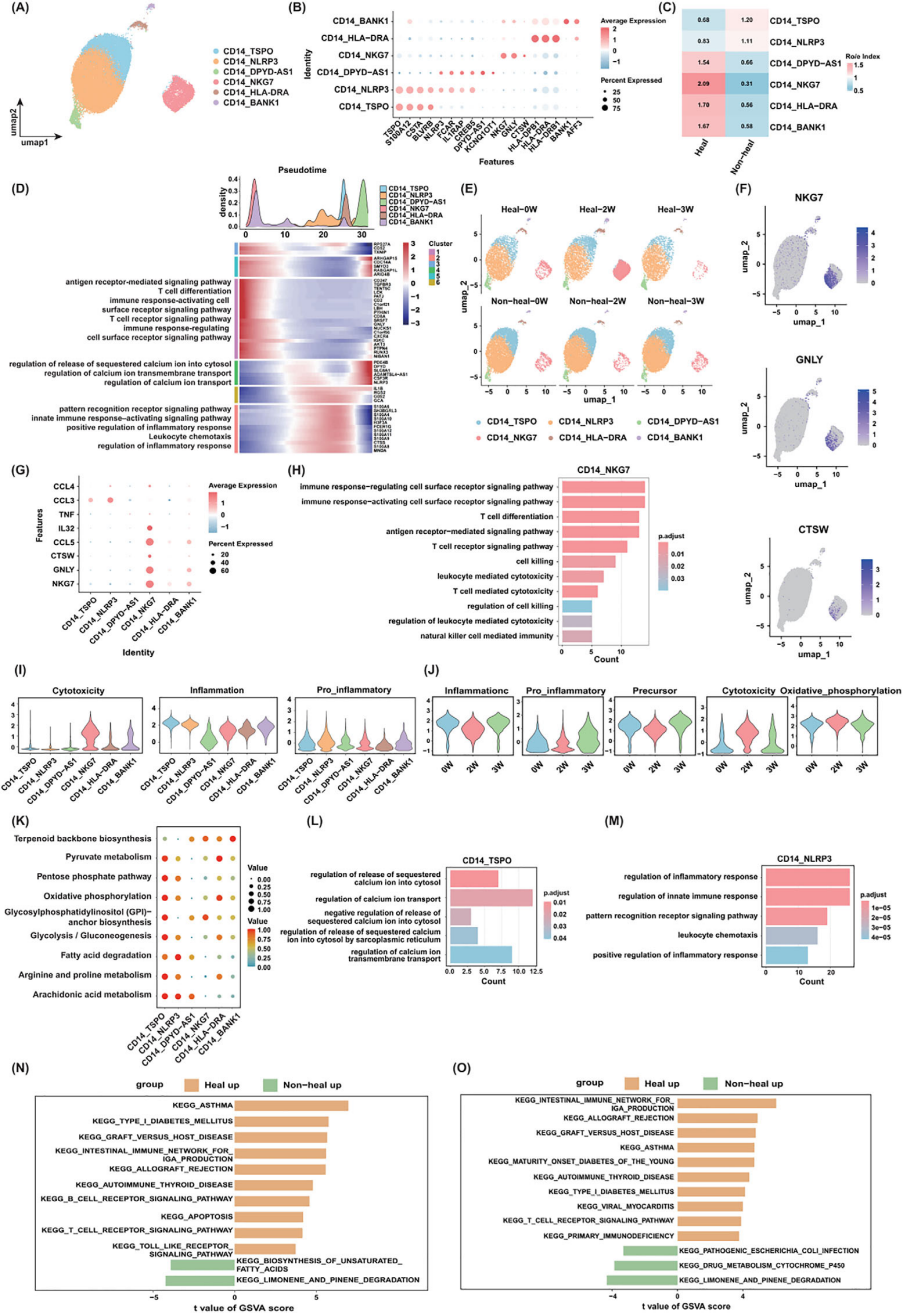
Single‐cell atlas of CD14^+^ monocytes in peripheral blood mononuclear cells (PBMCs) from diabetic foot ulcers (DFUs). (A) UMAP plot showing the distribution of different cell subsets of CD14^+^ monocytes. (B) Bubble plot depicting the expression of hallmark genes in the CD14^+^ monocyte subsets (detailed differential expression statistics are provided in Tables S4 and S5). (C) Ro/e analysis heatmap showing the expected expression differences of CD14^+^ monocyte subsets between the Heal and Non‐heal groups. (D) Pseudotime analysis showing the distribution and enriched pathways of CD14^+^ monocytes in pseudotime. (E) UMAP plot showing the distribution of CD14^+^ monocytes in different groups and at different treatment time points. (F) Expression of key genes in the CD14_NKG7 subset. (G) Dot plot showing the expression of cytotoxicity‐related genes and immune effector genes across CD14^+^ monocyte subsets. (H) Gene Ontology (GO) enrichment analysis of the CD14_NKG7 subset, highlighting cytotoxicity‐ and TCR signalling‐related pathways. (I) Violin plot showing the cytotoxic, inflammatory, and pro‐inflammatory functional phenotype scores of CD14^+^ monocyte subsets. (J) Violin plot showing the key functional phenotype scores of the CD14_NKG7 subset. (K) Key metabolic pathways enriched in CD14^+^ monocytes. (L) GO pathways enriched in CD14_TSPO. (M) GO pathways enriched in CD14_NLRP3. (N) Differential pathways enriched in CD14_TSPO in the Heal and Non‐heal groups. (O) Differential pathways enriched in CD14_NLRP3 in the Heal and Non‐heal groups.

Cytotoxicity genes (NKG7, GNLY and CTSW) in CD14_NKG7 monocytes peaked at 2 W, mirroring cell proportion kinetics (Figure 2F and Figure S2F,G), while immune effectors (CCL5, IL32, TNF, CCL3 and CCL4) showed significantly higher expression than in other CD14^+^ subsets (Figure 2G). Enriched GO terms included T cell‐ and leukocyte‐mediated cytotoxicity, cell killing, natural killer cell‐mediated immunity, and TCR signalling regulation (Figure 2H). At 2 W, cytotoxicity and oxidative phosphorylation scores peaked while inflammatory scores fell below baseline (Figure 2I,J). Collectively, CD14_NKG7 monocytes exhibit a cytotoxic‐like transcriptional signature and a “high‐efficacy, low‐inflammation” state. Notably, terpenoid backbone biosynthesis was markedly activated (Figure 2K); as SKHS‐derived terpenoids reportedly promote fibroblast migration and inhibit biofilm formation,^2^ this suggests a potential metabolic association with CD14_NKG7 dynamics. Finally, in an independent DFU PBMC scRNA‐seq dataset (GSE165816), the CD14_NKG7 signature was significantly higher in CD14^+^ monocytes from healers than non‐healers (*p* = 2.80 × 10^−8^; Figure S6F; Section S1.7).

In stark contrast, the Non‐heal‐enriched CD14_TSPO and CD14_NLRP3 subsets exhibited a “high‐energy, high‐oxidative” transcriptional state. CD14_TSPO was enriched in endoplasmic reticulum calcium transport (Figure 2L), whereas CD14_NLRP3 favoured pattern recognition receptor signalling activation (Figure 2M). Metabolic pathway analysis revealed that both subsets co‐activated glycolysis/gluconeogenesis, oxidative phosphorylation, and the pentose phosphate pathway (Figure 2N,O). Notably, this profile may relate to SKHS efficacy heterogeneity: in the Non‐heal group, these subsets upregulated limonene and pinene degradation alongside cytochrome P450 drug‐metabolism genes (Figure 2K and Figure S2H). Although these data cannot directly quantify local wound‐site metabolism, and given the abundance of monoterpenes in SKHS, we hypothesise that Non‐heal patients may harbour a systemic metabolic background favouring accelerated drug clearance^3^

CellChat analysis revealed that CD14_TSPO and CD14_NLRP3 formed dense interaction networks with T cells and NK cells (Figure 3; Figures S3 and S4). Dominated by CCL and VISFATIN signalling‐related ligand–receptor pairs, these interactions peaked at 2 W. This finding suggests that the CCL5–CCR1 axis and VISFATIN signalling may be involved in maintaining DFU immune imbalance.

**FIGURE 3:**
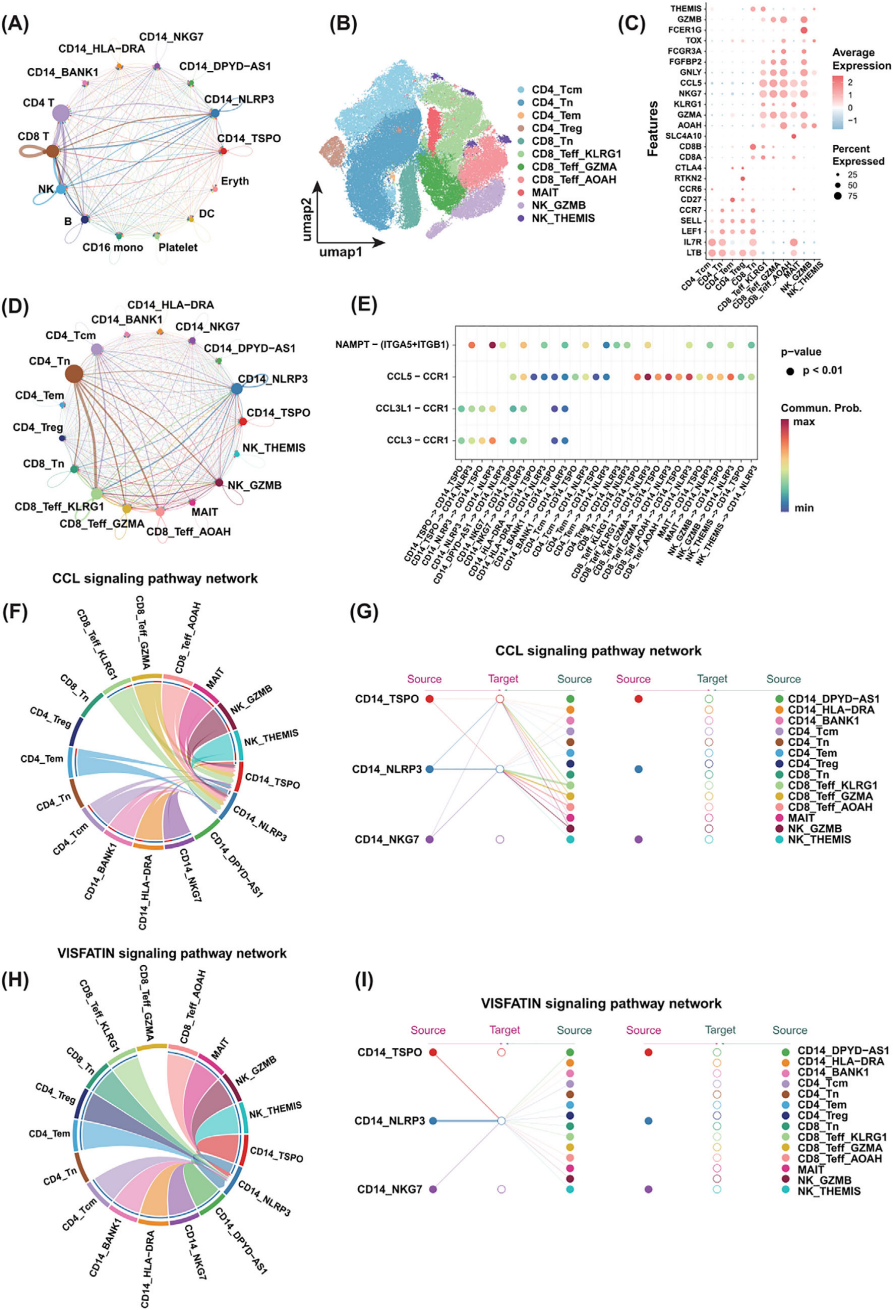
CellChat analysis of CD14^+^ monocytes in diabetic foot. (A) CellChat network diagram showing the interaction between CD14^+^ monocyte subsets and other cell types, with different colours and connections representing the relationships between immune cell subsets. (B) UMAP plot showing the distribution of T/NK cell subsets. (C) Bubble plot displaying the expression of key genes in the T/NK cell subsets. (D) CellChat network diagram showing the interaction between CD14^+^ monocyte subsets and T/NK cell subsets. (E) Bubble plot showing the probability of interaction between CD14^+^ monocyte subsets and T/NK cells in key signalling pathways. (F) CCL signalling pathway network diagram showing the interaction between CD14^+^ monocyte subsets and T/NK cell subsets in the CCL signalling pathway. (G) CCL cytokine‐receptor signalling pathway network diagram showing the signal transmission between key CD14^+^ monocyte subsets and other immune cell subsets in cytokine and receptor interactions. (H) VISFATIN signalling pathway network diagram showing the interaction between CD14^+^ monocyte subsets and T/NK cell subsets in the VISFATIN signalling pathway. (I) VISFATIN cytokine‐receptor signalling pathway network diagram showing the signal transmission between key CD14^+^ monocyte subsets and other immune cell subsets in cytokine and receptor interactions.

To explore the mechanisms underlying these phenotypes and metabolic reprogramming, SCENIC analysis constructed a TF‐target regulatory network (Figure 4 and Figure S5A). In the Non‐heal group, FOS family activity was associated with the “persistent inflammation–metabolic stress” phenotype. Specifically in CD14_TSPO monocytes, FOS activity paralleled TSPO expression and the enrichment of oxidative phosphorylation and monoterpene degradation, and co‐occurred with CCR1 upregulation (Figure S5B,E). In CD14_NLRP3 monocytes, FOSB activity correlated with NLRP3 expression and the enrichment of detoxification and cytochrome P450 pathways (Figure S5C,F). In contrast, the Heal‐enriched CD14_NKG7 subset showed elevated RUNX3, ETS1, and TBX21 activity, whose regulons included cytotoxic genes (NKG7 and GNLY) and, for RUNX3, terpenoid biosynthesis genes (Figure S5D,G). As SKHS is rich in reportedly anti‐inflammatory and anti‐biofilm terpenoids (e.g. gardenoside), this RUNX3‐associated program may further link CD14_NKG7 to therapeutic response.

**FIGURE 4:**
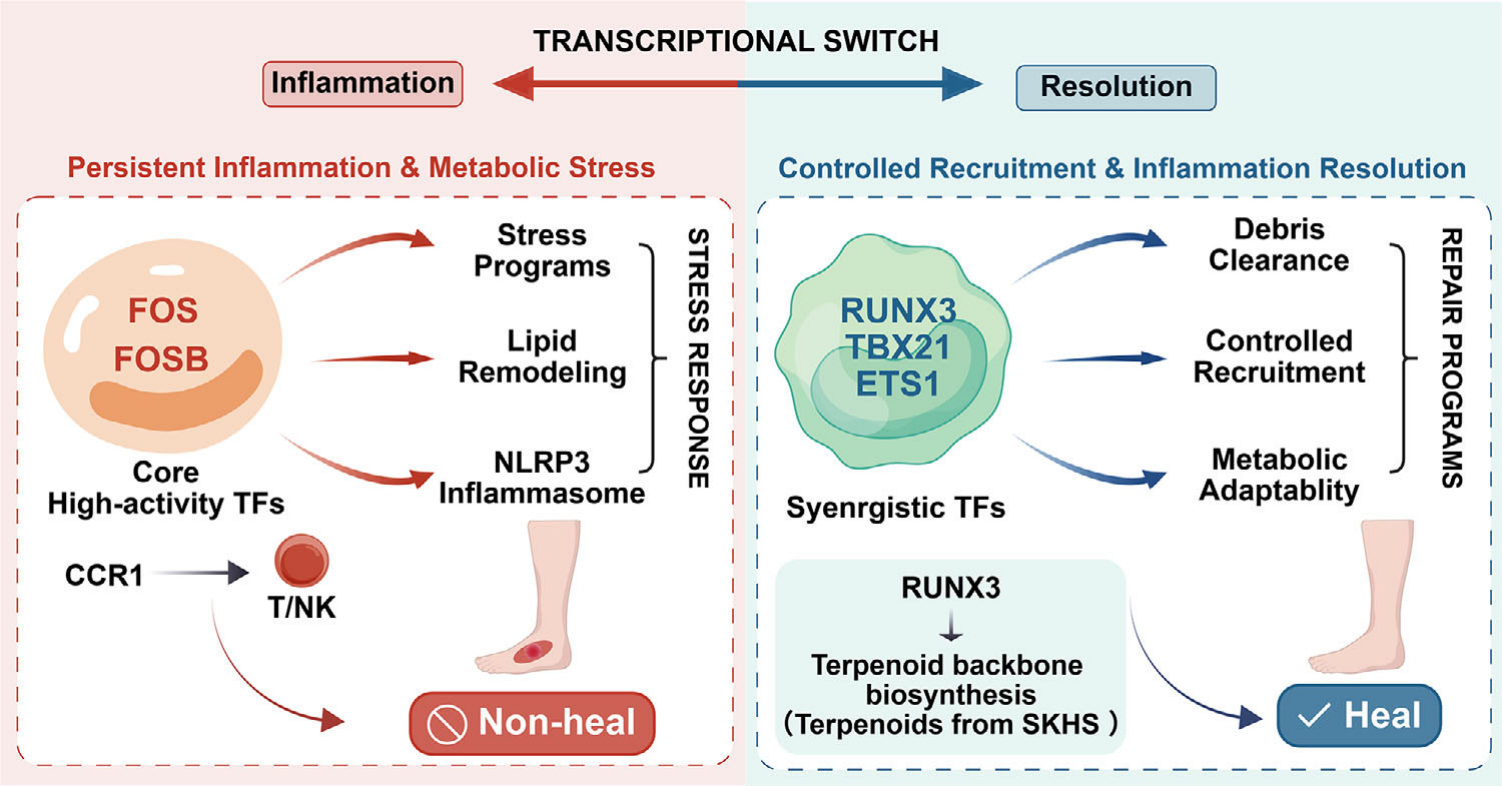
Transcription factor regulatory network in CD14^+^ monocytes. Created with BioGDP^4^.

In summary, longitudinal single‐cell transcriptomics reveals peripheral CD14^+^ monocyte dynamics in SKHS‐treated DFU patients, suggesting that healing involves both inflammation resolution and metabolic reprogramming. The CD14_NKG7 subset, with its cytotoxic‐like transcriptional signature and “high‐efficacy, low‐inflammation” state, is an accessible candidate biomarker. Conversely, Non‐heal monocytes show FOS/FOSB‐associated inflammatory programs, upregulated drug metabolism and enriched CCL5–CCR1 and VISFATIN signalling. Limitations include the small cohort (*n* = 5); these transcriptome‐based associations require confirmation in larger cohorts and functional experiments, and the single‐timepoint external dataset cannot validate the two‐week trajectory. Nevertheless, as peripheral monocytes are major precursors of wound‐infiltrating macrophages, these systemic alterations may illuminate the local wound immune microenvironment. Integrating matched tissue multi‐omics with emerging strategies such as negative pressure therapy^5^ and topical pharmacological interventions^6^, ^7^, ^8^ will accelerate precision DFU therapy.

## AUTHOR CONTRIBUTIONS

**Jue Zhang**: Conceptualisation, clinical experiments and data curation. **Wenyi Cao**: Data analysis and manuscript writing. **Mingxun Luo**: Assisted in methodology design and data processing. **Zhimin Gu**: Assisted in data analysis and visualisation. **Ming Mu**: Investigation and visualisation. **Junjiu Huang**: Supervision and review. **Xiaotong Luo**: Resource and funding acquisition, supervision and revision. **Aisheng Wei**: Supervision and revision.

## CONFLICT OF INTEREST STATEMENT

The authors declare no conflict of interest.

## ETHICS STATEMENT

This study was approved by the Ethics Committee of Foshan Hospital of Traditional Chinese Medicine (Approval No. KY【2022】041‐1) and conducted in accordance with the Declaration of Helsinki.

## PATIENT CONSENT STATEMENT

Written informed consent was obtained from all participants prior to enrolment.

## Supporting information



Supporting Information

Supporting Information

Supporting Information

## Data Availability

The datasets generated and/or analysed during the current study have been deposited in the Genome Sequence Archive (GSA) under accession number HRA017933. Additional data supporting the findings of this study are available from the corresponding author upon reasonable request.
