# Supplementary material for: Single‐cell transcriptomic profiling reveals transcription factor‐driven CD14+ monocyte programs underlying divergent wound healing outcomes in diabetic foot ulcers treated with Shangke Huangshui
Source: Clin Transl Med. 2026 Nov 14;16(11):e70838. doi: 10.1002/ctm2.70838 (PMC13770200; doi:10.1002/ctm2.70838)
Supplement: Supplementary file 1 [file CTM2-16-e70838-s001.docx]

**1 METHODS**

**1.1 Patient Recruitment and Ethical Statement**

This study was approved by the Ethics Committee of Foshan Hospital of Traditional Chinese Medicine (Approval No. [KY【2022】041-1]) and conducted in accordance with the Declaration of Helsinki. Patients diagnosed with DFUs categorized as Wagner grade 2, 3, or 4 were screened for eligibility (**Table S1**). A total of 20 prospective participants were enrolled after providing written informed consent, while individuals with active malignancies, severe systemic infections, or end-stage organ failure were excluded (**Table S2**).For the five patients included in the scRNA-seq analysis (Heal group, n=3; Non-heal group, n=2), baseline clinical characteristics were collected prior to SKHS treatment to assess the comparability of the two groups (**Table S3**), including age, sex, body mass index (BMI), duration of diabetes, duration of diabetic foot, Wagner grade, fasting blood glucose, white blood cell count, neutrophil percentage, and serum albumin. Continuous variables were compared using the Wilcoxon rank-sum test and categorical variables using Fisher's exact test.

**1.2 Clinical Treatment and Longitudinal Monitoring**

All participants received standardized standard-of-care (SOC) interventions, including surgical debridement, pressure offloading, infection control, and vascular assessment. In addition to SOC, patients were treated with topical SKHS following established clinical protocols. Clinical characteristics such as age, sex, body mass index (BMI), and duration of diabetes were documented at baseline. To track therapeutic progress, clinical parameters including blood glucose levels, complete blood counts (CBC), and immune factor assays were longitudinally monitored at baseline (0W), and at 2 weeks (2W) and 3 weeks (3W) post-treatment. Wound healing was further quantified through standardized photography and area measurements.

**1.3 Outcome Assessment and scRNA-seq Sub-sampling**

Following the 3-week follow-up, eight patients who completed the full protocol were evaluated for clinical outcomes. Patients were categorized based on the percentage of wound size reduction and re-epithelialization into a Heal group (n=5) and a Non-heal group (n=3). For high-resolution immune profiling, a representative subset of five patients was selected from this cohort, comprising three from the Heal group and two from the Non-Heal group. Frozen peripheral blood mononuclear cell (PBMC) samples from these five patients, collected at all three time points (0W, 2W, and 3W), were retrieved for subsequent longitudinal scRNA-seq analysis.

**1.4 Clinical Specimen Processing and Single-Cell Capturing**

Peripheral blood mononuclear cells (PBMCs) were isolated from 10 mL of anticoagulated whole blood using Ficoll-Paque PREMIUM density gradient centrifugation^1^. The diluted blood was carefully layered over the centrifugation medium and centrifuged at 2,000 rpm for 15 minutes without braking. The mononuclear cell layer was collected and washed twice with PBS containing 2% fetal bovine serum (FBS). Cell viability was assessed using trypan blue staining, and only samples with viability greater than 80% were used for downstream experiments. Single-cell capturing and library construction were performed using the 10x Genomics Chromium platform. Transcriptome libraries were prepared using the Chromium Single Cell 3′ Reagent Kits (v3). Single-cell capturing and library construction were performed using^2^.

**1.5 Computational Pipeline and Bioinformatic Analysis**

Raw sequencing data were processed using the Seurat (v4.0) R package^3^. Low-quality cells were filtered if they had fewer than 200 detected genes or over 5% mitochondrial content. To reduce batch effects, we applied the Harmony algorithm after independent sample identification^4^. We normalized the data, selected 2,000 highly variable genes, and scaled the counts. During scaling, we regressed out mitochondrial content to minimize technical noise. Dimensionality reduction was performed via Principal Component Analysis (PCA) and visualized using Uniform Manifold Approximation and Projection (UMAP). Unsupervised clustering was executed using the Louvain algorithm (resolution = 0.3)^5^. Cell-type identities were assigned by integrating FindAllMarkers-derived signatures with canonical lineage markers.The Observed/Expected (RO/e) ratio and AddModuleScore were employed to assess subset enrichment and inflammatory programs, respectively^6^. To evaluate specific biological states, we calculated inflammatory feature scores and other functional program activities for each cluster using the Seurat AddModuleScore function.

We characterized the functional states of identified clusters using multiple computational frameworks. clusterProfiler was used for Gene Ontology (GO) and KEGG enrichment analyses to identify active biological programs^7^. To assess pathway activity at the sample level, we applied a pseudo-bulk strategy to construct expression matrices, followed by GSVA scoring based on KEGG gene sets from the MSigDB database^8-11^. Metabolic heterogeneity was delineated using the scMetabolism framework. Specifically, we used the AUCell algorithm and KEGG metabolic gene sets to calculate activity scores at single-cell resolution.^12,13^.

To reconstruct developmental trajectories, we applied the Monocle2 DDRTree algorithm using significant ordering genes (q < 0.01)^14^. We independently assessed differentiation potential with CytoTRACE to validate pseudotime directionality. Intercellular communication was modeled using CellChat to identify signaling hubs. Finally, we inferred gene regulatory networks (GRNs) via pySCENIC. The resulting regulon activities were quantified and visualized using Cytoscape^15^.

Cell–cell communication was systematically evaluated using CellChat and the CellChatDB.human database^16^. Gene regulatory networks (GRNs) were inferred using the pySCENIC workflow^17^. Regulon activity was quantified using the AUCell algorithm. Key transcription factors and their target genes were identified for each subpopulation, and core TF–target regulatory networks were visualized using Cytoscape^18^.

**1.6 Statistical Analysis**

Statistical analyses were performed using R software (version 4.4.2). Continuous variables are presented as mean ± standard deviation (SD) or median (interquartile range, IQR), as appropriate. Intergroup differences were assessed using Student’s t-test or the Wilcoxon rank-sum test, depending on data distribution. Multiple comparisons in differential expression and functional enrichment analyses were corrected using the Benjamini–Hochberg method to control the false discovery rate (FDR). All statistical tests were two-sided, and P < 0.05 was considered statistically significant.

**1.7 External Validation Using an Independent DFU Dataset**

To assess the reproducibility of the CD14_NKG7 transcriptional signature and its association with DFU healing outcomes, an independent public DFU PBMC single-cell transcriptomic dataset (GSE165816; 11 DFU patients: 7 Healers, 4 Non-healers) was used for external validation. In this dataset, PBMCs were collected at a single time point at surgical debridement, corresponding to the 0W time point of our cohort, and healing outcomes were determined by 12-week post-operative follow-up. The dataset was processed following the same analytical pipeline described above: major immune cell types were annotated, and all CD14^+^ monocytes were extracted for sub-clustering. The cluster corresponding to the CD14_NKG7 profile was identified by the expression of NKG7, GNLY, and CTSW, and subset enrichment between groups was evaluated using the Ro/e ratio. To quantify the CD14_NKG7 transcriptional signature, a scoring module was constructed with the Seurat AddModuleScore function from the top 10 marker genes of CD14_NKG7 identified in our cohort, and module scores were compared between the Heal and Non-heal groups across all CD14^+^ monocytes. In parallel, module scores were compared between the two groups within the CD14_NKG7-like cluster (cluster 4). Between-group differences were assessed using the Wilcoxon rank-sum test.

**2 Supplementary Table**

**Table S1** Wagner classification of diabetic foot

| **Wagner Grade** | **Clinical Presentation** |
| --- | --- |
| 0 | Intact skin; may have deformity, neuropathy, or high-risk foot |
| 1 | Ulcer limited to the skin surface, no deep tissue involvement |
| 2 | Ulcer extends to tendon, bone, or joint capsule, without abscess or osteomyelitis |
| 3 | Presence of abscess, osteomyelitis, or joint sepsis |
| 4 | Gangrene limited to toes or a portion of the foot |
| 5 | Entire foot or major portion affected; usually requires amputation |

**Table S2** Patient inclusion and exclusion criteria

| **Inclusion Criteria** | **Exclusion Criteria** |
| --- | --- |
| 1. Patients aged ≥18 and <80 years.  2. Preliminary diagnosis of diabetic foot (DF) according to WHO criteria.  3. Provision of written informed consent. | 1. Neurodegenerative diseases (e.g., Parkinson’s disease, Alzheimer’s disease).  2.Cardiovascular diseases (e.g., heart failure, uncontrolled hypertension).  3.Chronic viral infections (e.g., viral hepatitis, HIV infection).  4. Pressure ulcers, venous ulcers, or arterial insufficiency–related ulcers.  5. Other severe medical conditions (e.g., malignancy, immunodeficiency).  6. Pregnancy or lactation.  7.Unhealthy lifestyle habits (e.g., smoking, excessive alcohol consumption). |

**Table S3** Baseline clinical characteristics of the participants included in the single-cell analysis.

| **Variable** | **Heal(n=3)** | **Non-heal(n=2)** | **Pvalue** |
| --- | --- | --- | --- |
| Age, years | 72.00 (54.00-83.00) | 62.50 (60.00-65.00) | 0.8 |
| Female sex, n/N | 2/3 (66.7) | 2/2 (100.0) | 1 |
| BMI, kg/m2 | 21.03 (17.73-24.20) | 26.25 (21.80-30.70) | 0.4 |
| Diabetes duration, years | 13.00 (7.00-20.00) | 10.00 (3.00-17.00) | 0.8 |
| DF/DFU duration, months | 3.00 (3.00-36.00) | 3.25 (0.50-6.00) | 0.7671 |
| Wagner grade >=3, n/N | 2/3 (66.7) | 2/2 (100.0) | 1 |
| Postprandial glucose, mmol/L | 7.29 (6.32-8.20) | 10.97 (10.35-11.60) | 0.2 |
| Fasting glucose, mmol/L | 6.90 (3.97-21.03) | 5.66 (4.80-6.52) | 0.8 |
| WBC, x10^9/L | 7.61 (7.58-10.72) | 20.45 (17.80-23.10) | 0.2 |
| Neutrophil % | 0.72 (0.64-0.74) | 0.87 (available n=1) | NA |
| Albumin, g/L | 34.95 (31.00-38.90) (available n=2) | 27.25 (26.00-28.50) | 0.3333 |

Abbreviations: BMI, body mass index; DF, diabetic foot; DFU, diabetic foot ulcer; WBC, white blood cell.

**3 Supplementary Figure**

**FIGURE S1** Quality Control and Features of PBMCs in Diabetic Foot Ulcers (DFU）(A)Violin plot displaying the distribution of nFeature_RNA, nCount_RNA, and percent.mt after quality control for different samples.
(B) UMAP plot showing the distribution of cells across different samples and treatment time points.
(C) Stacked bar chart illustrating the distribution of different cell populations in the Heal and Non-heal groups, as well as the distribution of cells across different samples.
(D) Ro/e analysis heatmap presenting the expected expression differences of cell populations at treatment time points 0W, 2W, and 3W between the Heal and Non-heal groups.
(E) Violin plot displaying the inflammatory functional phenotype scores of each cell population at treatment time points 0W, 2W, and 3W.

**FIGURE S2** Characteristics of CD14⁺ monocytes in PBMCs from diabetic foot ulcers (DFU)
(A) Differential pathways enriched in CD14⁺ monocytes in the Heal and Non-heal groups.
(B) Ro/e analysis heatmap showing the expected expression differences of CD14⁺ monocyte subsets between the Heal and Non-heal groups at 0W, 2W, and 3W treatment time points.
(C) Distribution of CD14⁺ monocyte subsets in pseudotime.
(D) CytoTRACE analysis showing the differentiation state of CD14⁺ monocyte subsets.
(E) Violin plot showing the functional scores of the CD14⁺ monocyte precursor phenotype.
(F) Expression of key genes NKG7, GNLY, and CTSW in the CD14_NKG7 in the Heal and Non-heal groups.
(G) Expression of key genes NKG7, GNLY, and CTSW in the CD14_NKG7 at treatment time points 0W, 2W, and 3W.
(H) UMAP plot showing the expression of key metabolic pathways in CD14⁺ monocyte.

**Figure S3** Interaction between CD14⁺ monocyte subsets and T/NK cell subsets .

**Figure S4** Interaction between CD14⁺ monocyte subsets and T/NK cell subsets at different treatment time points.
(A) CellChat network illustrating the interaction strength between CD14⁺ monocyte subsets and T/NK cell subsets at treatment time 0W.
(B) CellChat network showing the interaction strength between CD14⁺ monocyte subsets and T/NK cell subsets at treatment time 2W.
(C) CellChat network demonstrating the interaction strength between CD14⁺ monocyte subsets and T/NK cell subsets at treatment time 3W.
(D) CCL and VISFATIN cytokine-receptor signaling pathway network at treatment time 0W, highlighting key CD14⁺ monocyte subsets and their signaling interactions with other immune cell subsets.
(E) CCL and VISFATIN cytokine-receptor signaling pathway network at treatment time 2W, illustrating key CD14⁺ monocyte subsets and their signaling interactions with other immune cell subsets.
(F) CCL and VISFATIN cytokine-receptor signaling pathway network at treatment time 3W, depicting key CD14⁺ monocyte subsets and their signaling interactions with other immune cell subsets.

**Figure S5** Key transcription factors in CD14⁺ monocyte subsets.
(A) Bubble plot illustrating the expression levels of key transcription factors across CD14^+^ monocyte subsets.
(B) Top 10 specific transcription factors for CD14_TSPO subset, highlighting their significance in regulating this monocyte subset.
(C) Top 10 specific transcription factors for CD14_NLRP3 subset, emphasizing their regulatory roles in this monocyte subset.
(D) Top 10 specific transcription factors for CD14_NKG7 subset, focusing on their contribution to the function of this monocyte subset.
(E) Transcription factor regulatory network for CD14_TSPO subset constructed using Cytoscape, depicting key regulatory relationships.
(F) Transcription factor regulatory network for CD14_NLRP3 subset constructed using Cytoscape, illustrating the interactions between transcription factors and their targets.
(G) Transcription factor regulatory network for CD14_NKG7 subset constructed using Cytoscape, showing the intricate regulation of this monocyte subset.

**Figure S6** External validation of the CD14_NKG7 transcriptional signature using an independent DFU PBMC dataset (GSE165816).

(A) UMAP plot displaying the distribution of major immune cell types in the external dataset.

(B) Bubble plot showing the expression of canonical marker genes across cell types.

(C) UMAP plot displaying sub-clusters of CD14+ monocytes.

(D) Dot plot showing the expression of cytotoxicity-related genes (NKG7, GNLY, CTSW) across CD14+ monocyte clusters.

(E) Ro/e analysis heatmap displaying the enrichment of CD14+ monocyte clusters between the Heal and Non-heal groups.

(F) Violin plot showing the CD14_NKG7 signature score across all CD14+ monocytes between the Heal and Non-heal groups (p = 2.80×10-8).

(G) Violin plot showing the CD14_NKG7 signature score within cluster 4 between the Heal and Non-heal groups (p = 0.33, ns).

**REFERENCES**

1. Böyum A. Isolation of mononuclear cells and granulocytes from human blood. Isolation of monuclear cells by one centrifugation, and of granulocytes by combining centrifugation and sedimentation at 1 g. Scand J Clin Lab Invest Suppl. 1968;97:77-89.
2. Zheng, G., Terry, J., Belgrader, P. et al. Massively parallel digital transcriptional profiling of single cells. Nat Commun 8, 14049 (2017). <https://doi.org/10.1038/ncomms14049>
3. Hao Y, et al. Integrated analysis of multimodal single-cell data. Cell. 2021;184(13):3573–3587.e29.
4. Korsunsky, I., Millard, N., Fan, J. et al. Fast, sensitive and accurate integration of single-cell data with Harmony. Nat Methods 16, 1289–1296 (2019). <https://doi.org/10.1038/s41592-019-0619-0>
5. Blondel VD, Guillaume JL, Lambiotte R, Lefebvre E. Fast unfolding of communities in large networks. J Stat Mech. 2008;2008(10):P10008. doi:10.1088/1742-5468/2008/10/P10008
6. Schürch CM, Bhate SS, Barlow GL, et al. Coordinated cellular neighborhoods orchestrate antitumoral immunity at the colorectal cancer invasive front. Cell. 2020;182(5):1341-1359.e19. doi:10.1016/j.cell.2020.07.005
7. Yu G, Wang LG, Han Y, He QY. clusterProfiler: an R package for comparing biological themes among gene clusters. OMICS. 2012;16(5):284-287. doi:10.1089/omi.2011.0118
8. Yu G, Wang LG, Han Y, He QY. clusterProfiler: an R package for comparing biological themes among gene clusters. OMICS. 2012;16(5):284-287. doi:10.1089/omi.2011.0118
9. Hänzelmann S, Castelo R, Guinney J. GSVA: gene set variation analysis for microarray and RNA-seq data. BMC Bioinformatics. 2013;14:7. doi:10.1186/1471-2105-14-7
10. Subramanian A, Tamayo P, Mootha VK, et al. Gene set enrichment analysis: a knowledge-based approach for interpreting genome-wide expression profiles. Proc Natl Acad Sci U S A. 2005;102(43):15545-15550. doi:10.1073/pnas.0506580102
11. Kanehisa M, Goto S. KEGG: Kyoto Encyclopedia of Genes and Genomes. Nucleic Acids Res. 2000;28(1):27-30. doi:10.1093/nar/28.1.27
12. Wu Y, Yang S, Ma J, et al. Spatiotemporal immune landscape of colorectal cancer liver metastasis at single-cell level. Cancer Discov. 2022;12(1):134-153. doi:10.1158/2159-8290.CD-21-0316
13. Aibar S, González-Blas CB, Moerman T, et al. SCENIC: single-cell regulatory network inference and clustering. Nat Methods. 2017;14(11):1083-1086. doi:10.1038/nmeth.4463
14. Qiu X, Mao Q, Tang Y, Wang L, Chawla R, Pliner HA, Trapnell C. Reversed graph embedding resolves complex single-cell trajectories. Nat Methods. 2017;14(10):979-982. doi:10.1038/nmeth.4402
15. Gulati GS, Sikandar SS, Wesche DJ, et al. Single-cell transcriptional diversity is a hallmark of developmental potential. Science. 2020;367(6476):405-411. doi:10.1126/science.aax0249
16. Jin S, Guerrero-Juarez CF, Zhang L, et al. Inference and analysis of cell-cell communication using CellChat. Nat Commun. 2021;12(1):1088. doi:10.1038/s41467-021-21246-9
17. Aibar S, González-Blas CB, Moerman T, et al. SCENIC: single-cell regulatory network inference and clustering. Nat Methods. 2017;14(11):1083-1086. doi:10.1038/nmeth.4463
18. Shannon P, Markiel A, Ozier O, et al. Cytoscape: a software environment for integrated models of biomolecular interaction networks. Genome Res. 2003;13(11):2498-2504. doi:10.1101/gr.1239303
